# Supplementary material for: Atomic sawtooth-like metal films for vdW-layered single-crystal growth
Source: Nat Commun. 2024 Jul 11;15:5848. doi: 10.1038/s41467-024-50184-5 (PMC11239812; doi:10.1038/s41467-024-50184-5)
Supplement: Supplementary file 1 — Supplementary Information [file 41467_2024_50184_MOESM1_ESM.pdf]

Supplementary Information

Atomic sawtooth-like metal films for vdW-layered single-crystal growth

Hayoung Ko[1†], Soo Ho Choi[1,2†], Yunjae Park[3†], Seungjin Lee[1], Chang Seok Oh[1], Sung Youb Kim[3,4], Young Hee Lee[1,2*], Soo Min Kim[5*], Feng Ding[6,7*], and Ki Kang Kim[1,2*]

Affiliations:

[1]*Department of Energy Science, Sungkyunkwan University (SKKU), Suwon 16419, Republic of Korea*

[2]*Center for Integrated Nanostructure Physics (CINAP), Institute for Basic Science (IBS), Sungkyunkwan University (SKKU), Suwon 16419, Republic of Korea*

[3]*Graduate School of Carbon Neutrality, Ulsan National Institute of Science and Technology (UNIST), Ulsan 44919, Republic of Korea*

[4]*Department of Mechanical Engineering, Ulsan National Institute of Science and Technology (UNIST), Ulsan 44919, Republic of Korea*

[5]*Department of Chemistry, Sookmyung Women's University, Seoul, 14072, Republic of Korea*

[6]*Department of Materials Science and Engineering, Ulsan National Institute of Science and Technology (UNIST), Ulsan 44919, Republic of Korea*

[7]*Shenzhen Institute of Advanced Technology, Chinese Academy of Science, Shenzhen, China*

*Correspondence to: leeyoung@skku.edu, soominkim@sookmyung.ac.kr, f.ding@siat.ac.cn, kikangkim@skku.edu

† These authors are equally contributed.

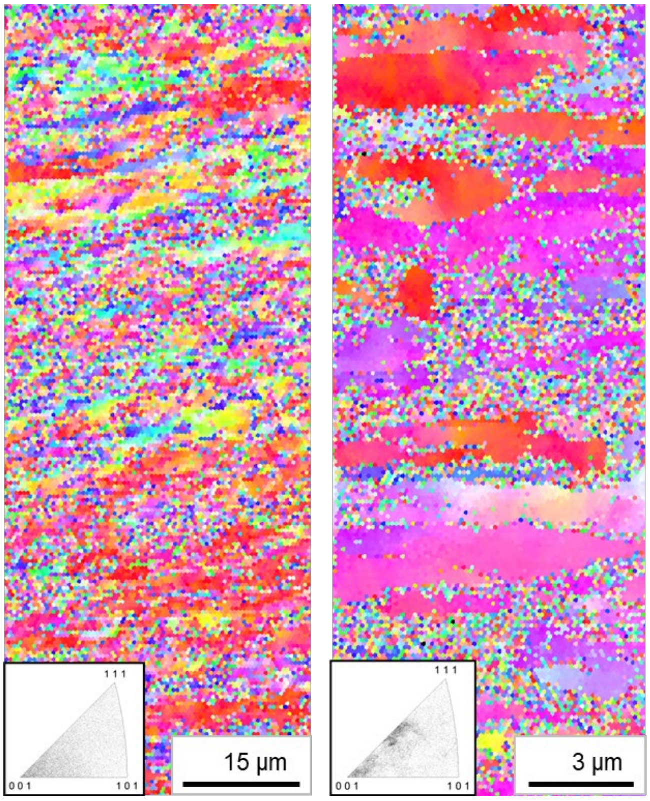

Fig. S1 EBSD mapping images and IPF maps of as-received PC W foils in various regions.

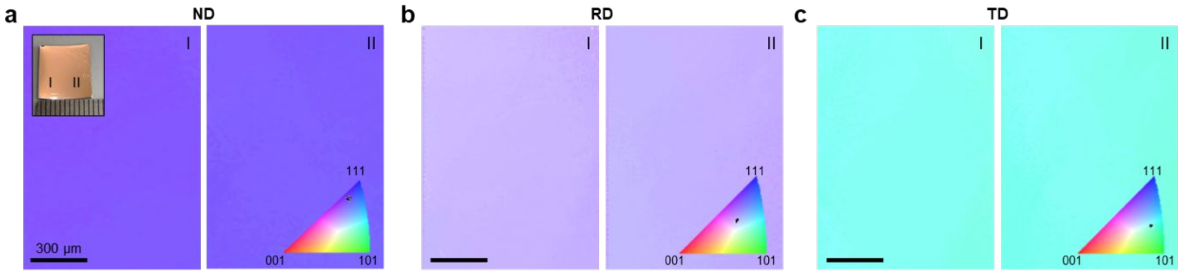

Fig. S2 EBSD mapping images of SC AS Cu film. The EBSD mapping images were obtained at positions (I and II) along the **a**, normal direction (ND), **b**, rolling direction (RD), and **c**, transverse direction (TD), respectively. The inset shows a photograph of a centimeter-scale SC AS Cu substrate.

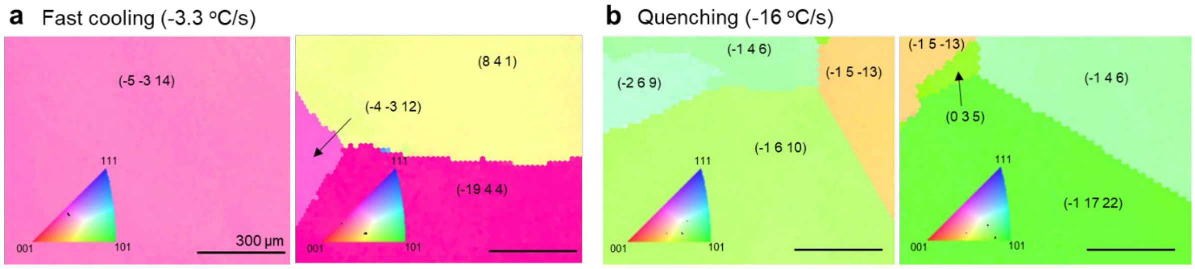

Fig. S3 EBSD images with IPF maps of Cu surfaces after MS process with different cooling rates. a, fast cooling (-3.3 °C/s), and **b,** quenching (-16 °C/s) at two different regions.

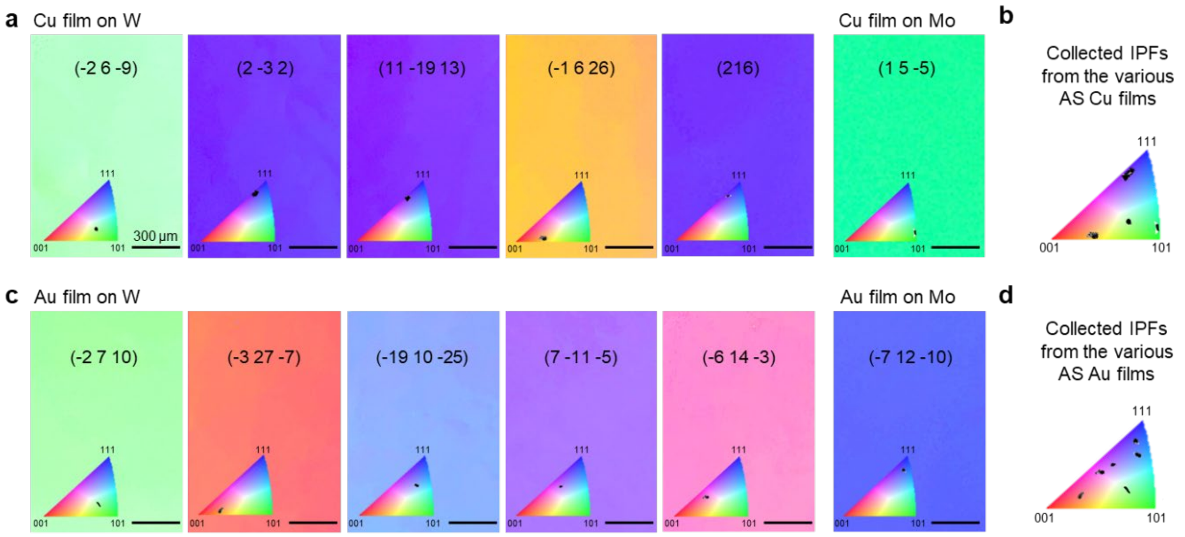

Fig. S4 EBSD mapping images and IPF maps obtained for each SC AS. **a**, Cu and **c**, Au films on W and Mo foils. **b,d**, IPFs collected from the samples in **a** and **c**.

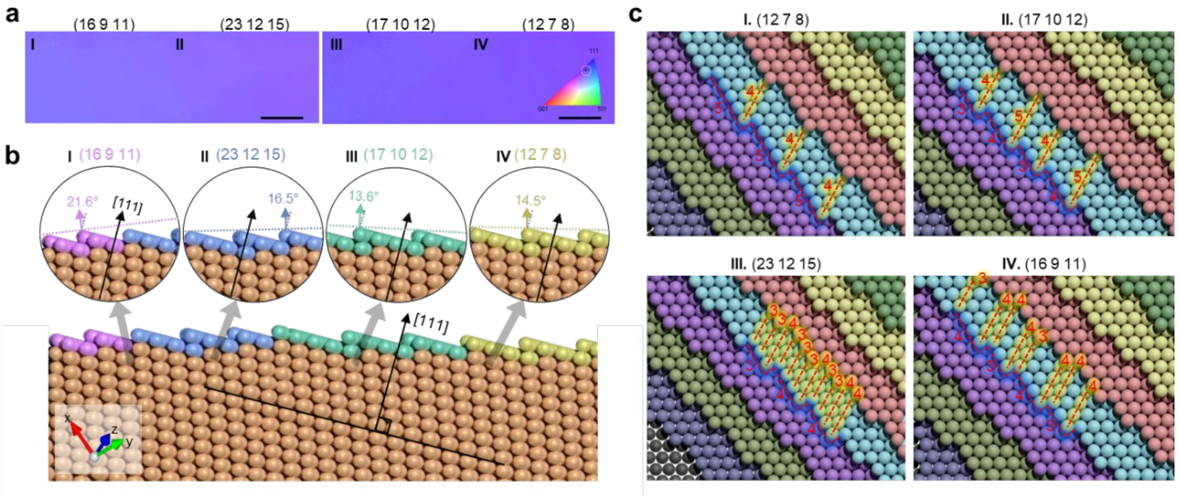

Fig. S5 Detailed analysis of SC AS Cu films. a, EBSD mapping images of the SC AS Cu film. **b**, Cross-section and **c**, top-view atomic structures corresponding to each Miller index in **a**.

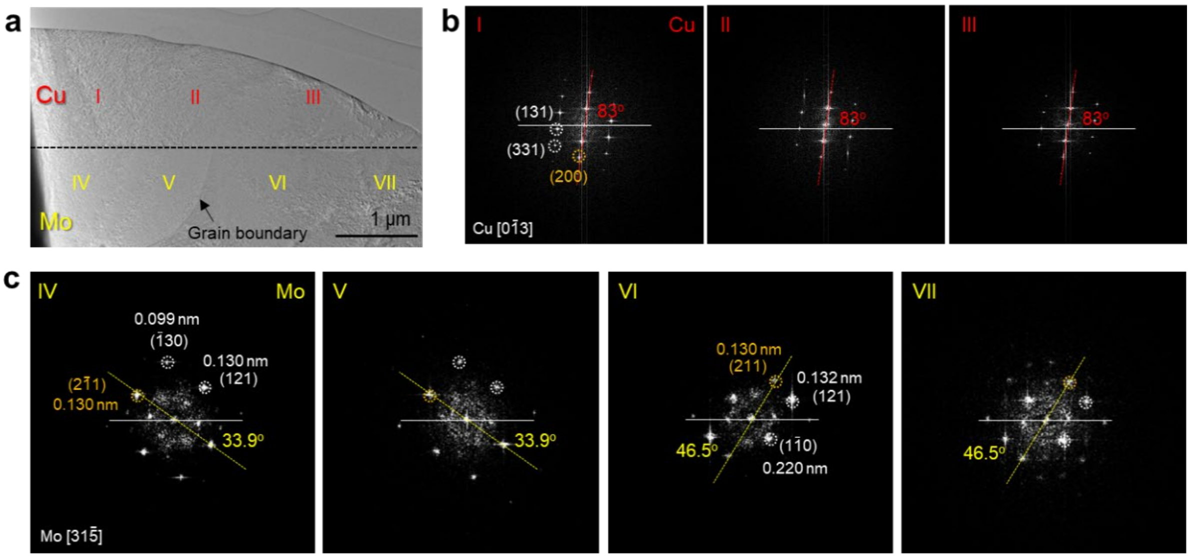

Fig. S6 Cross-sectional TEM analysis of SC Cu film on PC Mo foil. a, Low-magnification cross-sectional TEM image of Cu/Mo interface. **b,c**, FFT patterns obtained from various regions indicated by the Roman numerals in **a**.

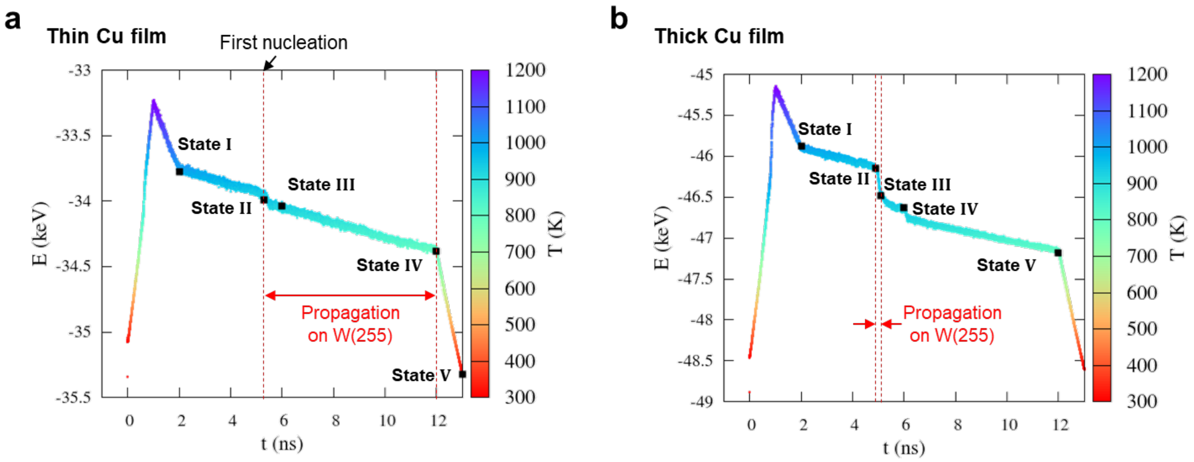

Fig. S7 System energy changes in the MD simulations for **a,** thin and **b,** thick Cu films on a PC W surface. The states indicate each phase change in Figures 3b and c.

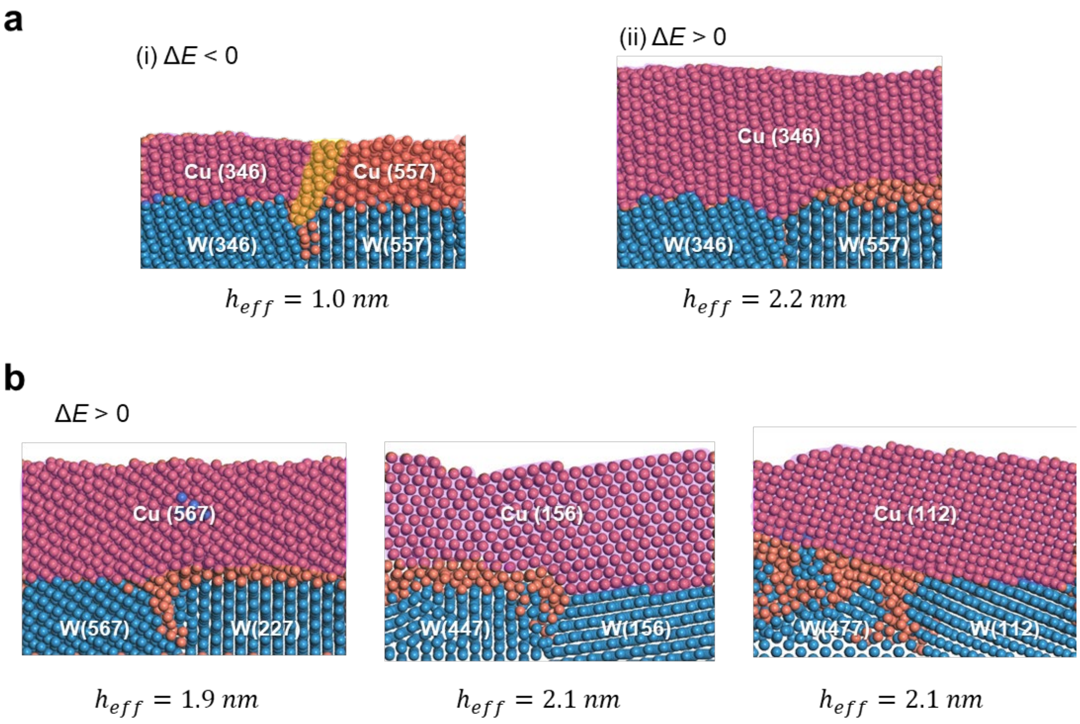

Fig. S8 MD simulation results **a**, for the Cu/W system on the W(346) and W(557) grains with different Cu thicknesses (h_{eff}: effective thickness of Cu) and **b**, for other Cu/W systems with various W grains.

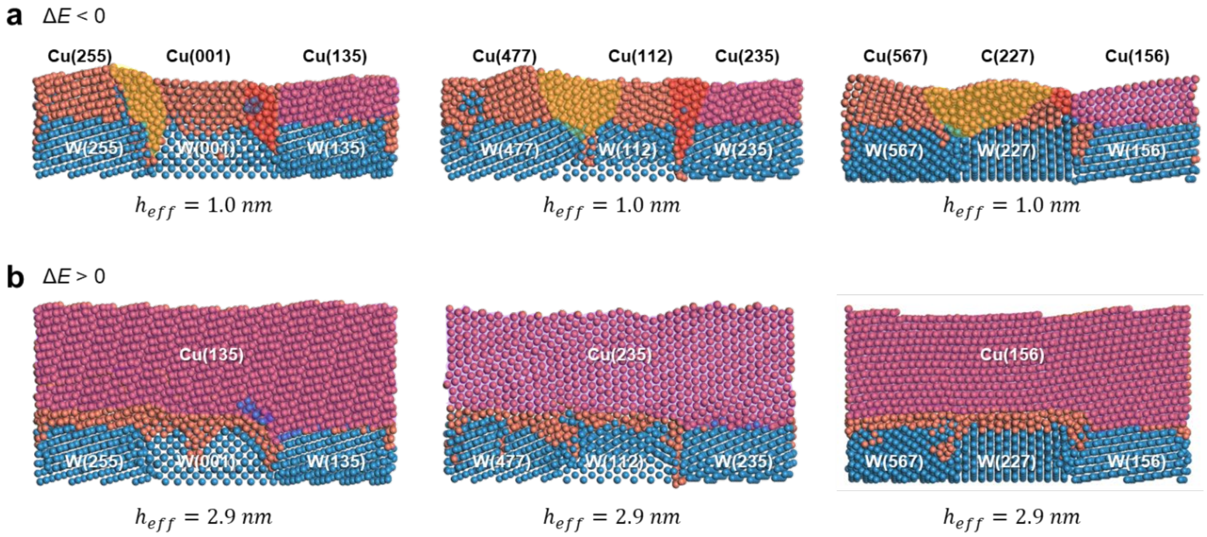

Fig. S9. MD simulation results of Cu/W system with different three W indices. Simulated results about different three W indices with **a,** thin Cu and **b,** thick Cu.

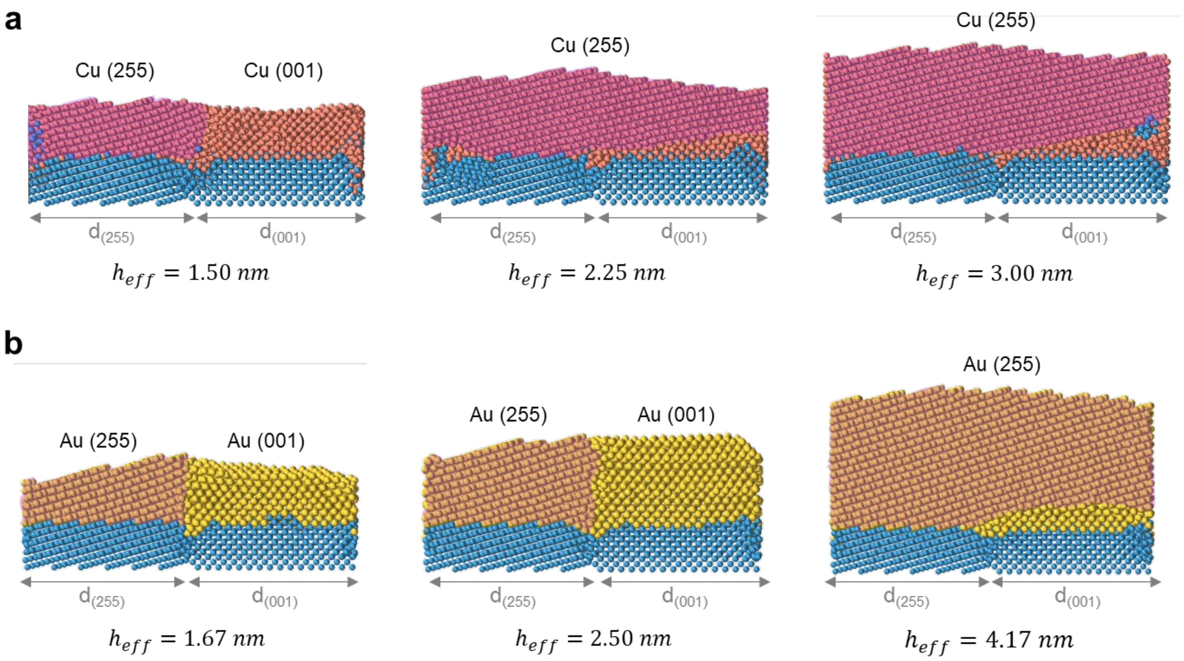

Fig. S10 MD simulation results for **a**, Cu/W and **b**, Au/W systems with various Cu and Au thicknesses (h_{eff}: effective height of Cu and Au).

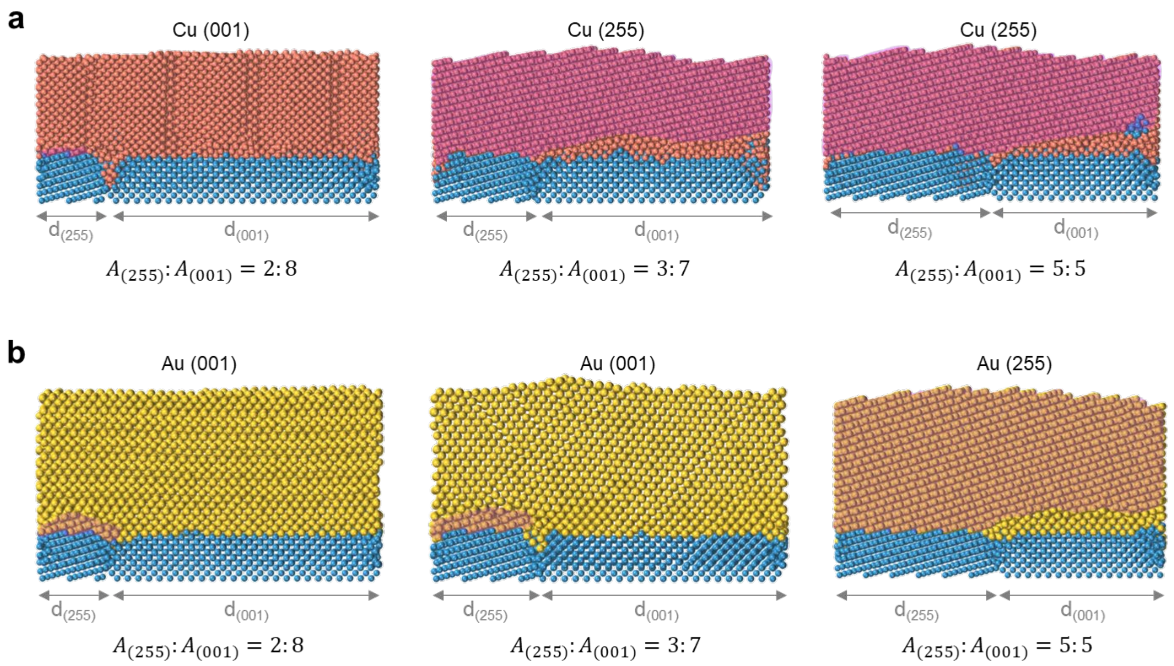

Fig. S11 MD simulation results for **a**, Cu/W and **b**, Au/W systems with various areal ratios of W(255) to W(001) grains (d: grain size, A: surface area).

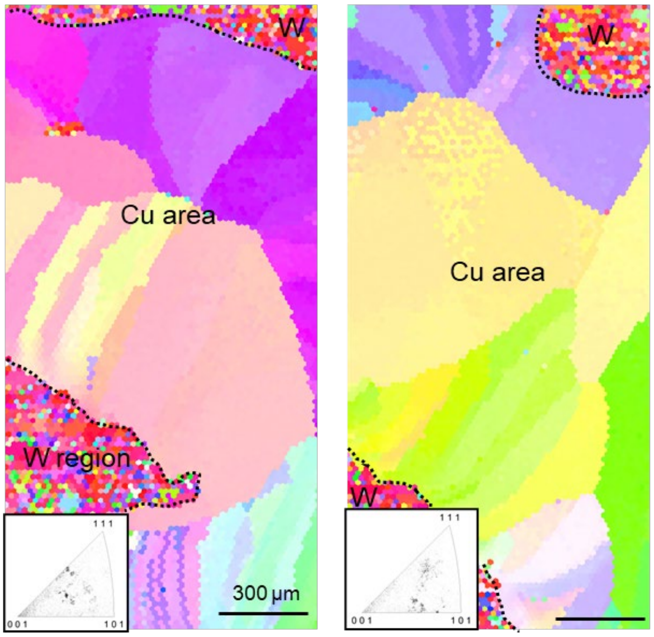

Fig. S12 EBSD mapping images and IPF maps of solidified 3-μm-thick Cu foil on a PC W foil.

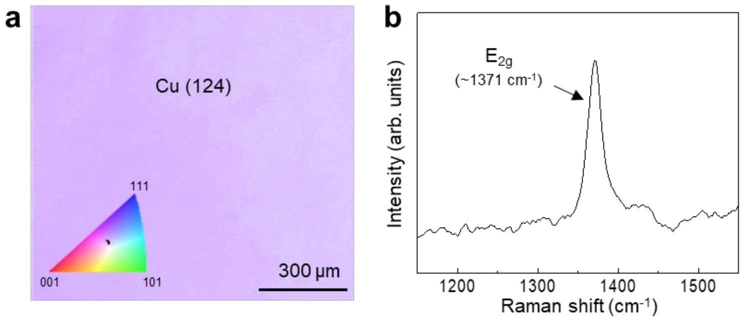

Fig. S13 Growth of SC hBN on SC AS Cu substrate. a, EBSD mapping image of an SC AS Cu film after growth of the SC hBN film. **b**, Representative Raman spectrum of SC hBN after the transfer onto a SiO_2/Si substrate.

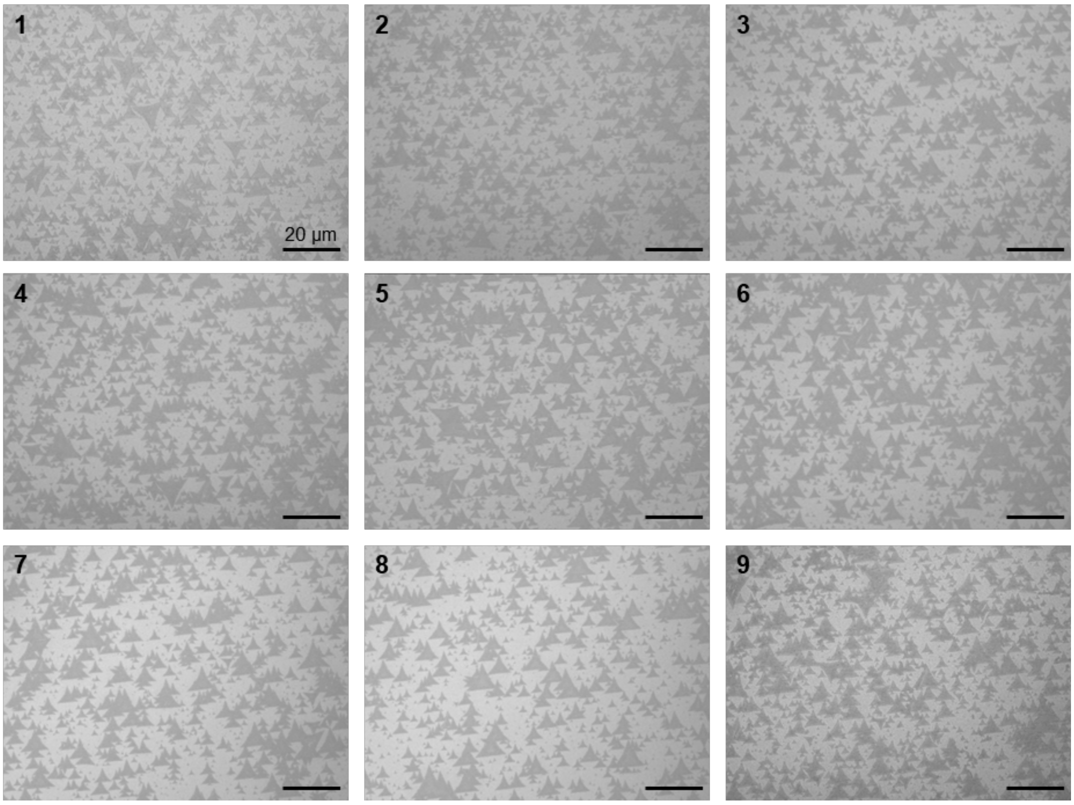

Figure S14 | SEM images of aligned hBN grains on a centimeter-scale SC AS Cu film. In total, 96.53% of the hBN grains are coherently aligned over the entire region.

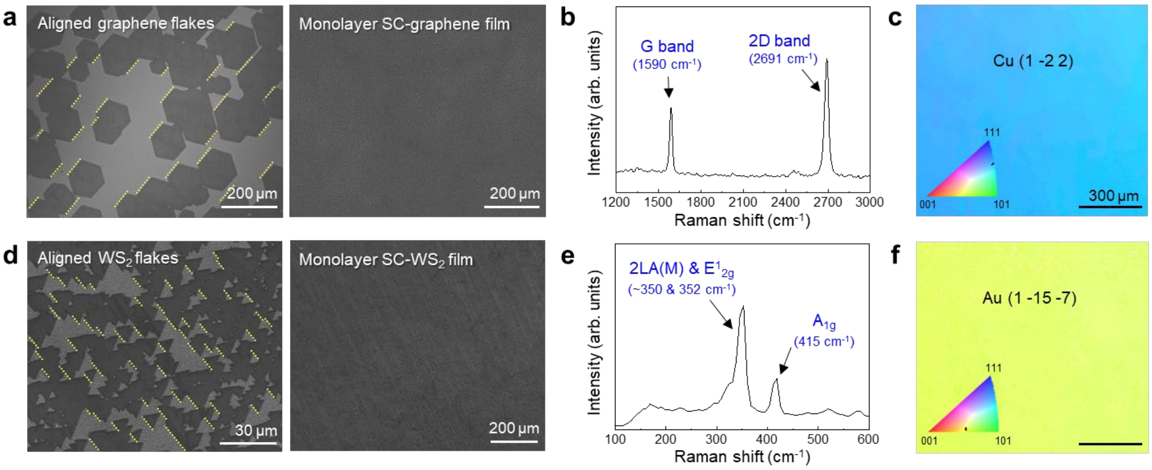

Figure S15 | Growth of SC graphene and WS_2 on SC AS Cu and Au films. a,d, SEM images of coherently aligned graphene, WS_2 grains, and SC films grown on the SC AS Cu and Au films. **b,e**, Representative Raman spectra of SC graphene and WS_2 films. **c,f**, EBSD-mapping images of the SC AS Cu and Au films.
